# Comparative Analysis of Temperature and Stress Simulations in Mass Concrete for Sluice Gate Structures Based on Chinese and American Standards

**DOI:** 10.3390/ma18010100

**Published:** 2024-12-30

**Authors:** Jiaming Zhang, Dan Zhou, Xiangyu He, Xing Hu, Sheng Qiang

**Affiliations:** 1 College of Water Conservancy and Hydropower Engineering, Hohai University, Nanjing 210098, China; zjming0110@163.com (J.Z.); hxyu2021@163.com (X.H.); 2 NHRI R & D Tech Group Co., Ltd., Nanjing 210029, China; danzhou2006@163.com; 3 Shanghai Investigation, Design & Research Institute, Shanghai 200335, China; 4 China Railway Water Conservancy &Hydropower Planning and Design Group, Nanchang 330029, China; hustar0101@163.com

**Keywords:** secondary development, temperature control, civil engineering standards, finite element modeling

## Abstract

Temperature-induced cracks during the construction of large concrete structures, such as water gates and bridges, caused by hydration heat, pose a serious threat to structural safety and reliability. To address this, various countries have developed temperature control standards and guidelines for mass concrete structures, providing design direction and evaluation criteria. China and the United States (U.S.) are leaders in the field of temperature control for mass concrete structures, with significant influence in international projects. The study of the differences in temperature control results between the two countries’ standards not only helps to understand the impact of different regulations on temperature control design but also provides more design options for multinational projects. This study uses ABAQUS software (version 2023)to establish a finite element model of a water gate and employs secondary development of ABAQUS to simulate the temperature and stress fields under both Chinese and U.S. standards. The results indicate that the overall temperature and stress distributions of the structure are similar under both standards. The calculation results for internal and surface characteristic points show that the maximum temperature predicted by the Chinese standard is slightly higher than that of the U.S. standard, with a difference of no more than 1.7 °C. However, the maximum tensile stress calculated under the Chinese standard is lower than that of the U.S. standard, with internal stress differences not exceeding 0.23 MPa and surface stress differences not exceeding 0.56 MPa. This study provides a direct comparison of the temperature control results between the two standards, offering valuable insights for international projects to balance cost and safety, while also providing empirical evidence for optimizing national standards.

## 1. Introduction

Mass concrete is widely used in large-scale infrastructure projects, such as dams, sluices, and bridges, providing high load-bearing capacity and long-term structural stability that are crucial to project safety and reliability [1,2,3]. However, the definition of mass concrete varies internationally. According to the Chinese Standard GB50496-2018 [4], the British Standard BS882 [5], and the German Standard DIN1045 [6], mass concrete refers to concrete structures with a minimum dimension of at least 1 m, in which temperature changes due to hydration and shrinkage may cause detrimental cracking. In contrast, the American Standard ACI 116R [7] defines mass concrete as “large-scale concrete requiring measures to control the cement hydration heat and volume changes to reduce cracking risk.” The Japan Architectural Society Standard (JASS) specifies mass concrete as concrete with a minimum cross-sectional dimension exceeding 80 cm and an internal hydration-induced temperature rise of over 25 °C compared to the external environment [8]. Despite these differences, all countries emphasize the necessity of temperature control for mass concrete.

During the placement of mass concrete, substantial hydration heat rapidly raises the internal temperature [9,10], while the elastic modulus remains low and creep is significant, resulting in relatively low compressive stresses. As the structure cools over time, the elastic modulus increases and creep decreases, leading to tensile stresses under constraint [11,12,13], which may cause temperature-induced cracking [14,15]. Once cracks form, carbonation accelerates, affecting the stress distribution and load-bearing capacity, thus compromising the structure’s stability and durability. This degradation can lead to operational failures and economic losses [16]. To mitigate the adverse effects of hydration heat, various countries have developed temperature control standards for mass concrete to guide assessments and design.

China is the world’s largest developer and exporter of hydropower, with extensive domestic hydropower infrastructure and active participation in international projects under the “Belt and Road” initiative [17]. The U.S., a pioneer in hydropower development, has established widely recognized temperature control standards that are applied globally, offering invaluable engineering and management expertise [18]. Given the increasing collaboration in cross-national hydropower projects, comparing temperature control simulations under Chinese and U.S. standards provides scientifically validated design options that balance safety and cost-efficiency. Such comparisons also identify potential areas for improvement in temperature control simulations, offering insights for technical advancements.

The differences in temperature control simulation calculations between the Chinese and American codes are primarily reflected in the different standards for core calculation parameters (such as surface heat dissipation coefficient, creep, adiabatic temperature rise, etc.), which will be discussed in detail in Section 2. Furthermore, there are differences in the temperature control standards for massive concrete temperature fields specified in the two codes. Specifically, the Chinese standard considers the damaging effects of high temperatures on concrete structures due to the dehydration and decomposition of ettringite, and thus sets a clear upper limit for the center temperature of massive concrete, which should not exceed 80 °C. Additionally, the temperature difference between the interior and exterior of the concrete should not exceed 25 °C [4]. In contrast, the American standard does not provide specific numerical limits but proposes a calculation formula for the concrete pouring temperature, as shown in Equation (1), based on the consideration that tensile stress generated during the process of temperature drop from peak to final stable temperature may exceed the strain capacity of the concrete. The standard also discusses the specific effects of cooling, noting that, in massive concrete structures, for every 6 °C reduction in the pouring temperature below the average ambient temperature, the maximum temperature that the concrete will reach will decrease by approximately 3 °C [7].

(1) $$T_{i}=T_{f}+\frac{100\times C}{e_{t}\times R}-A_{t}$$

In Equation (1), $T_{i}$ is placing temperature of concrete; $T_{f}$ is final stable temperature of concrete; C is strain capacity (in millionths); $e_{t}$ is coefficient of thermal expansion per deg of temperature (in millionths); R is degree of restraint (in percent); and $A_{t}$ is initial temperature rise of concrete.

Moreover, the American code explicitly points out that, due to the lower elastic modulus and higher creep rate of early-age concrete, significant stress and strain will not develop during the concrete setting period and the short time immediately after setting. Therefore, the code suggests that surface cooling measures (such as laying cooling pipes) should continue for 2 to 3 weeks after concrete pouring, thereby creating a higher temperature gradient on the surface of the concrete. During this time, the concrete surface, constrained by internal temperature differences, generates compressive stress. This early compressive state helps reduce the formation of surface cracks and gradually relieves stress through the concrete’s creep effect, providing a buffer for subsequent tensile stresses caused by temperature fluctuations. However, the Chinese code does not mention this, but instead requires immediate surface insulation measures, such as laying thermal blankets, after the completion of concrete pouring, to prevent significant temperature gradients between the surface and the interior.

**Figure 1:**
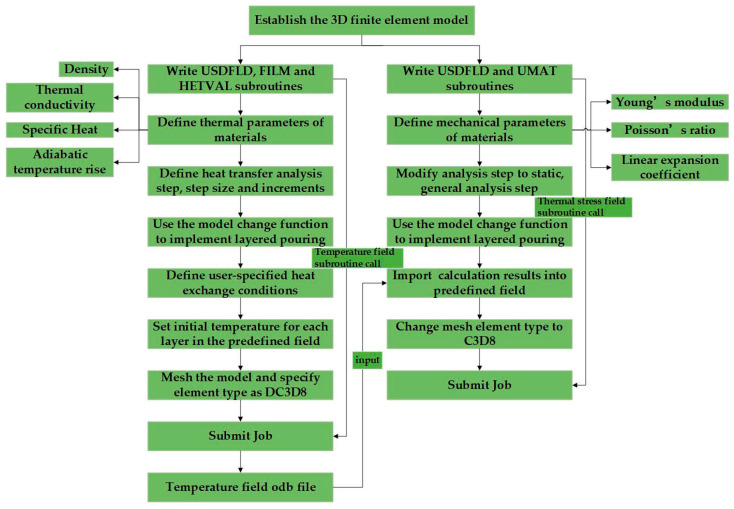
ABAQUS thermo-mechanical coupling step.

This study investigates the temperature and stress fields during the construction phase of a sluice in China, using simulations based on both Chinese and U.S. standards. The calculation process is shown in Figure 1. The simulation under the U.S. standard was conducted with a subroutine developed for ABAQUS, with the feasibility validation provided in the Appendix A. The results indicate that the temperature field and stress field calculations under both Chinese and American codes are similar, effectively reflecting the temperature and stress field variations during the early-age period and throughout the entire calculation duration of the sluice gate. The maximum temperature and the temperature difference between the surface and the interior calculated using the Chinese code are higher than those calculated using the American code, while the maximum tensile stress is lower. This difference is primarily due to the variations in the surface heat dissipation coefficient and creep calculation models between the two codes. The findings suggest that the Chinese code imposes stricter temperature control requirements, while the American code exhibits a higher sensitivity to stress control.

## 2. Calculation Parameters Under Chinese and U.S. Standards

### 2.1. Surface Heat Dissipation Coefficient

**Figure 2:**
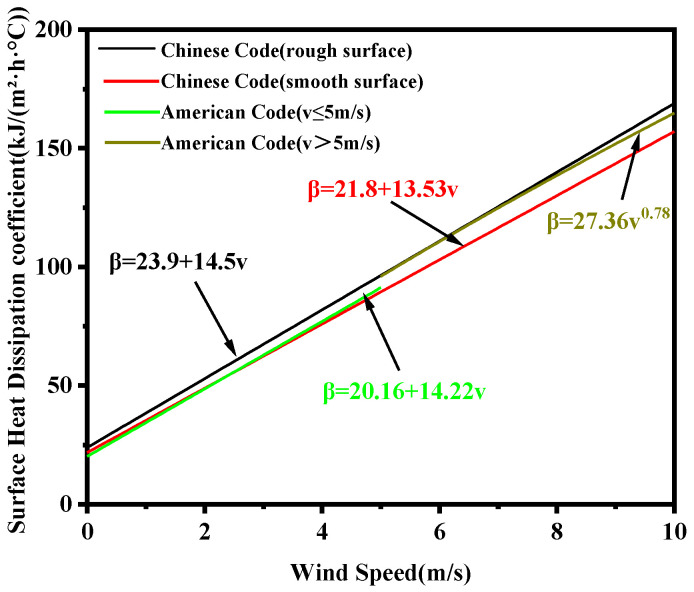
Surface heat dissipation coefficient calculation formulae in Chinese and American codes.

The surface heat dissipation coefficient represents the rate of heat transfer between the concrete surface and the external environment. It is a critical parameter in temperature control calculations for mass concrete and is influenced by multiple factors, such as surface roughness, wind speed, temperature difference, and insulation measures [19]. The Chinese standard [4] states that, when the concrete surface is directly exposed to air, the surface heat dissipation coefficient is linearly related to wind speed. The formulae for calculating the heat dissipation coefficient for rough and smooth surfaces are different. The American Standard ETL1110-2-54220 [20] specifies that the surface heat dissipation coefficient depends solely on wind speed and is independent of surface roughness. The calculation formulae for the surface heat dissipation coefficient under the Chinese and American standards are shown in Figure 2.

In Figure 2, ν is wind speed, m/s and β is the surface heat dissipation coefficient, kJ/(m^2^·h·°C).

### 2.2. Creep Model

According to the basic theory of elastic creep [21], the strain increment of concrete under complex stress conditions consists of elastic strain, creep strain, thermal strain, autogenous volume strain, and drying shrinkage strain. Based on extensive experimental research, Both China and the United States have each developed creep prediction models that align with engineering practices [9]. The commonly used creep models in the American Standard ACI 209R-92 [22] include the ACI 209 creep model [23], the B3 creep model [24], and the GL2000 creep model [25]. The calculation formulae for each model are shown in Table 1.

In Equation (2), t represents the concrete age, d; τ is the loading age of the concrete, d; (t−τ) denotes the duration of loading, d; E(τ) is the elastic modulus at the time of loading, MPa; $E_{0}$ represents the final value of the elastic modulus, MPa; and C(t,τ) is the creep coefficient of the concrete. In Equations (3)–(5), $J(t,t_{o})$ is the compliance function; $\phi 28(t,t_{o})$ is the creep coefficient; $E_{cmto}$ is the modulus of elasticity at the time of loading, MPa; $\gamma_{c}$ is the density of concrete, kg/m^3^; a and b are constants related to cement type and concrete curing type; Ψ is the time ratio related to the shape and size of the component; $\phi_{u}$ is the ultimate creep coefficient; $q_{1}$ is the instantaneous strain caused by unit stress; $C_{O}(t,t_{o})$ is the basic creep compliance; $C_{d}(t,t_{o},t_{c})$ is the drying creep compliance; $q_{2}\sim q_{5}$ are parameters related to the dimensions and material properties of the concrete; $t_{c}$ represents the drying age; $Q(t,t_{o})$ is the integral function related to the loading age of the concrete; H(t) is the function of relative pore humidity; $\chi (t,t_{0})$ is the aging coefficient;$f_{cm28}$ is the average compressive strength of concrete at 28 days, MPa; $f_{c}^{\prime }$ is the characteristic compressive strength of concrete, MPa; $f_{cmt}$ is the correction factor for compressive strength of concrete; and $E_{cmt}$ is the modulus of elasticity, MPa.

Among the above models, the ACI 209 model is significantly influenced by the size of the structural element and is less adaptable to the creep behavior of concretes with varying strength levels. The B3 model involves complex parameters and is cumbersome to apply. In contrast, the GL2000 model has fewer parameters, is simpler to calculate, and has a better fit to experimental data than the ACI 209 model, while being comparable to the B3 model [26]. Therefore, this study adopts the GL2000 creep model for stress field calculations under the American standard.

### 2.3. Adiabatic Temperature Rise Model

The heat of hydration is the primary cause of the rapid temperature rise in mass concrete, and the adiabatic temperature rise reflects the intensity of the concrete’s hydration heat. The Chinese standard includes several commonly used models to calculate adiabatic temperature rise, such as the exponential, hyperbolic, and composite exponential models [9]. The commonly used adiabatic temperature rise models in the U.S. include the Maximum Allowable Temperature Difference Method [27], the PCA Method [28], the ACI 207.2R Chart Method [29], and the Schmidt Temperature Gradient Method [30]. The calculation formulae for each model are shown in Table 2.

In Equations (6)–(8), θ(τ) is the adiabatic temperature rise at age τ, °C; $\theta_{0}$ is the final value of the hydration heat, °C; τ is the age, d; and m, n, a, b is a constant. In Equations (9)–(11), ΔT is the maximum temperature difference, °C; τ is the age, d; $\varepsilon_{tsc}$ is the tensile strain capacity of the concrete; K is the correction factor for sustained loading and creep deformation; R is the restraint factor; $\alpha_{c}$ is the thermal expansion coefficient of mature concrete; $T_{\max}$ is the maximum temperature of the concrete; g is the placement temperature of the concrete, °C; $T_{i}$ is the placement temperature of the concrete, °C; $W_{c}$ is the cement content per unit volume of concrete, kg/m^3^; $W_{scm}$ is the binder content per unit volume of concrete, kg/m^3^; $Q_{H}$ is the heat generation term, W/m^3^; ρ is the density of the concrete, kg/m^3^; $C_{p}$ is the specific heat, J/(kg·°C); α is the thermal conductivity of the concrete, W/(m·°C); Δt is the time step, s; and Δx is the distance between nodes, m.

In this study, the composite exponential model, proposed by Zhu Bofang, was adopted in the calculations based on the Chinese standard. This model demonstrates higher accuracy compared to the exponential and hyperbolic models [21] and the values of parameters a and b shown in Table 3.

**Figure 3:**
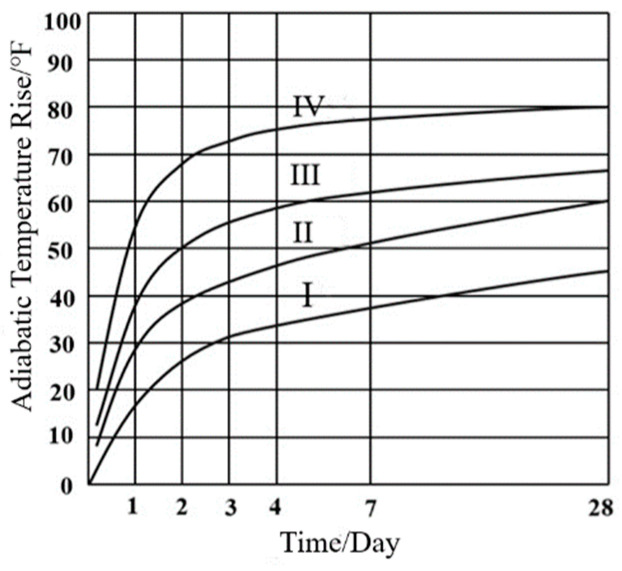
Adiabatic temperature rise curves of cements with different fineness.

In commonly used adiabatic temperature rise models in the United States, The Maximum Allowable Temperature Difference Method considers factors such as concrete tensile strain, thermal expansion coefficient, and cement type. It is simple to calculate but offers limited accuracy. The PCA Method, proposed by the Portland Cement Association, quickly estimates the peak temperature, although it does not account for the influence of fly ash and slag on hydration temperature rise. The ACI 207.2R Chart Method emphasizes that cement fineness has a greater influence on the heat generation rate than on total heat release. Increased fineness leads to a more intense hydration reaction and a faster temperature rise. Figure 3 shows the adiabatic temperature rise curves for different cement fineness. The hydration heat corresponding to different fineness of cement is shown in Table 4. However, as cement fineness and strength increase, the applicability of this curve to current mass concrete has decreased. The Schmidt Temperature Gradient Method uses a finite difference approach to calculate temperatures at discrete nodes, but its computational efficiency is lower. Given the substantial changes in construction methods, formwork types, cement fineness, supplementary cementitious materials (SCM), and chemical admixtures, these traditional methods are no longer adequate to meet the demands of modern mass concrete temperature field calculations. In this study, the adiabatic temperature rise model under the U.S. standard is consistent with the Chinese standard, adopting the composite exponential model.

## 3. Temperature and Stress Field Simulation

### 3.1. Project Overview

**Figure 4:**
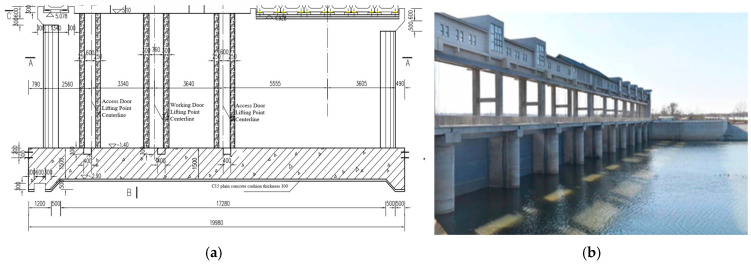
Tidal gate. (**a**) Schematic of the base slab and middle pier structure of the tidal gate; (**b**) completed tidal gate.

This study focuses on a tidal barrier located at the estuary of the Huanglei River in Weihai City, Shandong Province, China. The structural design of the tidal barrier is relatively complex, with a base slab measuring 20 m in length, 7.5 m in width, and 2 m in height, while the gate pier measures 18.7 m in length, 1.5 m in width, and 6.8 m in height. The cushion layer is set to a height of 0.1 m. The structural layout is shown in Figure 4. As a representative of mass concrete engineering, this tidal barrier is prone to significant temperature rises and thermal gradients during construction, which may lead to thermal cracking and other quality issues. The structure exemplifies the challenges commonly encountered in mass concrete projects. By conducting a numerical simulation of the temperature and stress fields during the construction period, this study aims to provide insights for the thermal control design of the tidal barrier and offer theoretical guidance for similar complex hydraulic mass concrete structures.

### 3.2. Calculation Parameters

This study uses the base slab and middle pier structure of the tidal barrier gate as the calculation model. The foundation consists of rock and soil foundations, with concrete piles in the soil foundation made of C30-grade concrete. The cushion layer beneath the middle pier slab is made of C15-grade concrete, while the base slab and pier are constructed with C35-grade concrete. The thermodynamic parameters of the foundation and the concrete used in the calculations are provided in Table 5.

### 3.3. Boundary Conditions

The annual average temperature data for Weihai City is shown in Table 6.

**Figure 5:**
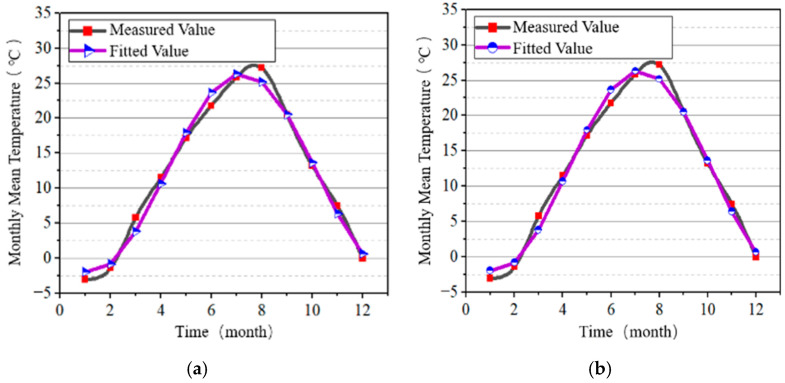
Comparison of fitted and measured temperatures in Weihai. (**a**) China code; (**b**) US code.

The cosine temperature function from both the Chinese and American standards was used to fit the measured temperature data, yielding monthly average temperature variation formulae for each standard, as shown in Equation (12), and daily average temperature variation formulae, as shown in Equation (13). The comparison between the fitted and measured values is shown in Figure 5.

(12) $$\left\{\begin{array}{l} T_{a}(t)=12.18+14.21\times \cos\left[\frac{\pi }{6}\left(t-7.2\right)\right]\\ T_{a}(t)=12.18-14.21\times \sin\left[\frac{\pi }{6}\left(t+1.8\right)\right] \end{array}\right.$$

(13) $$\left\{\begin{array}{l} {T_{a}}^{d}\left(\tau \right)=T_{a}(t)+6\times \cos\left[\frac{\pi }{12}\left(\tau -14\right)\right]\\ {T_{a}}^{d}\left(\tau \right)=T_{a}(t)-6\times \sin\left[\frac{\pi }{12}\left(\tau +4\right)\right] \end{array}\right.$$

In Equations (12) and (13), $T_{a}(t)$ represents the monthly average temperature, °C; ${T_{a}}^{d}\left(\tau \right)$ represents the daily average temperature, °C; t represents the month, month; and τ represents any given time within a 24-h day, h.

For the first 5 days of concrete curing, the surface is covered with steel formwork. Before the formwork is removed, the surface heat dissipation coefficient of the concrete is set to 400 kJ/(m^2^·d·°C). Once the formwork is removed, the surface heat dissipation coefficient is increased to 1497.36 kJ/(m^2^·d·°C). The initial temperature of the foundation is set to 18 °C, and the thermodynamic boundary conditions for the foundation are defined as adiabatic on the sides and bottom, with heat dissipation on the top surface, where the heat dissipation coefficient is 1497.36 kJ/(m^2^·d·°C). The mechanical boundary conditions are set to normal constraints on the sides and bottom, with other boundaries being free.

### 3.4. Construction Schedule

**Figure 6:**
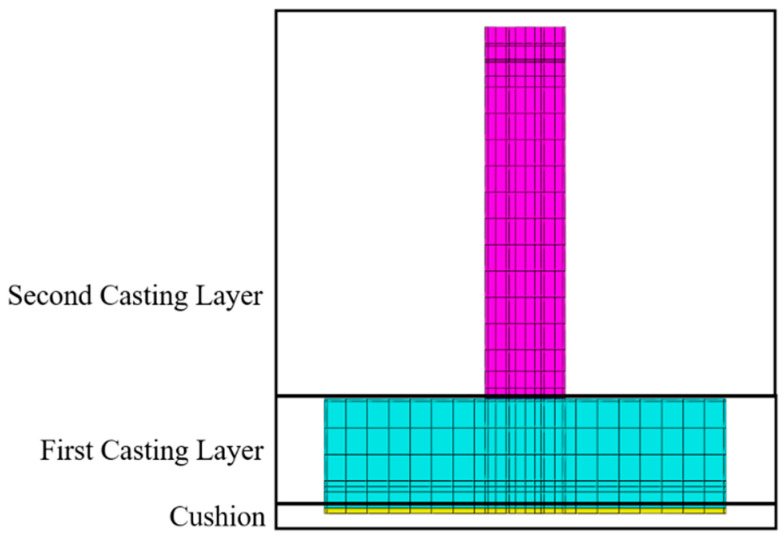
Schematic diagram of layered pouring for the tidal gate.

The construction of the sluice gate begins in winter, with a 10-day interval between the pouring of the base slab and the leveling layer, and a 110-day interval between the pouring of the base slab and the sluice pier. The pouring schedule for the sluice gate is shown in Table 7, and the construction sequence is depicted in Figure 6.

### 3.5. Finite Element Model

**Figure 7:**
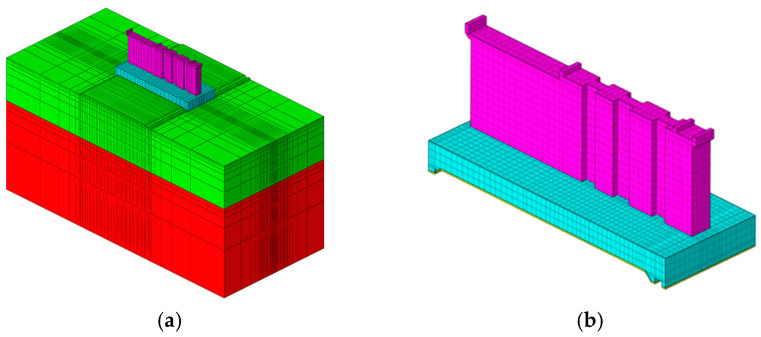
Finite element model. (**a**) Tidal sluice gate; (**b**) pier and slab.

The finite element model of the tide gate is shown in Figure 7, which fully considers the thermal and mechanical properties of the foundation and concrete. The coordinate system adopts a Cartesian coordinate system, where the *X*-axis is aligned perpendicular to the flow, the *Y*-axis follows the flow direction, and the *Z*-axis represents the elevation direction. In Figure 7a, the green section represents the soil foundation in contact with the cushion, while the red section represents the rock foundation. The foundation model extends 3 times the length of the sluice gate in the flow direction, 3.6 times its width in the transverse direction, and 3.4 times its height in the depth direction. After establishing the model, a mesh is generated, with finer meshing applied to the casting layers to ensure computational accuracy. The model contains a total of 53,940 elements and 60,023 nodes. The temperature and stress fields of the sluice gate are calculated using a sequential thermal–mechanical coupling method, with a simulation duration of 500 days.

### 3.6. Selection of Key Points

**Figure 8:**
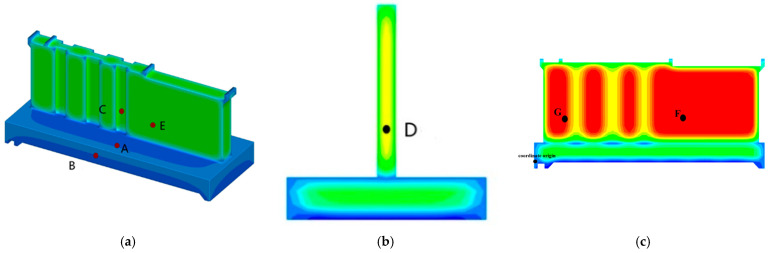
Key points’ locations. (**a**) Surface of the tidal sluice gate; (**b**) cross-section at Y = 9.7 m; (**c**) cross-section at X = 0 m.

To compare and analyze the temperature and stress fields at key locations of the water gate structure and typical cross-sections within the pier, seven characteristic points (A, B, C, D, E, F, G) on the slab and pier are selected for study. The coordinates of the characteristic points in the water gate model are detailed in Table 8, and their positions are shown in Figure 8.

## 4. Comparison of Calculation Results

### 4.1. Temperature Field Calculation Results

#### 4.1.1. Temperature Field Contour Plot

**Figure 9:**
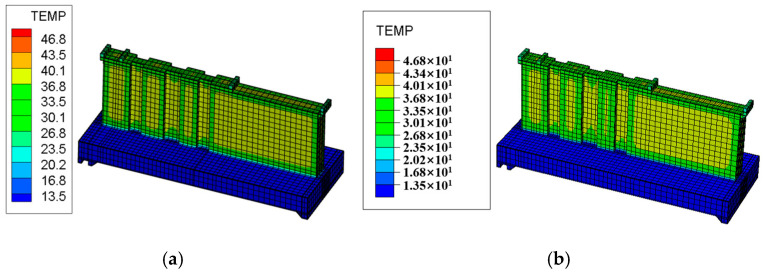
Temperature field contour map of the tidal barrier 1day after the pier pouring. (**a**) Chinese code; (**b**) U.S. code.

**Figure 10:**
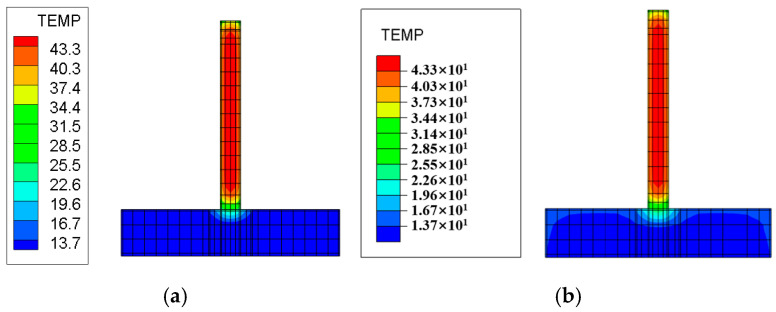
Temperature field contour map of the section at Y = 10 m, 1 day after pier pouring. (**a**) Chinese code; (**b**) U.S. code.

**Figure 11:**
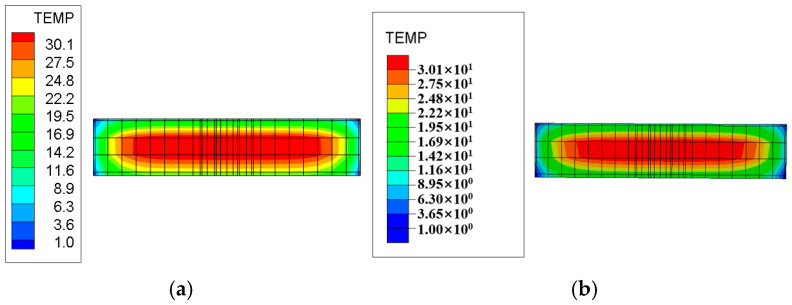
Temperature field contour map of the section at Y = 10 m, 5 days after slab pouring. (**a**) Chinese code; (**b**) U.S. code.

**Figure 12:**
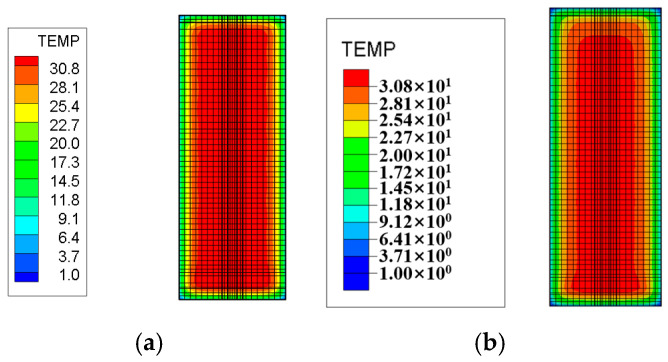
Temperature field contour map of the section at Z = 0.95 m, 5 days after slab pouring. (**a**) Chinese code; (**b**) U.S. code.

The layered construction conditions of the tidal sluice are simulated, and typical moments and sections from the finite element calculations are selected to compare the maximum temperature and its distribution under both Chinese and American standards. The temperature field contour maps for the surface and interior of the tidal sluice gate model are shown in Figure 9, Figure 10, Figure 11 and Figure 12.

As shown in Figure 9, the maximum surface temperature of the pier is approximately 40 °C under both the Chinese and U.S. codes. As indicated in Figure 11 and Figure 12, the high-temperature region of the slab, calculated according to the Chinese code, has a slightly larger distribution area compared to the U.S. code. The internal maximum temperature and the temperature difference between the surface and interior for each typical section are listed in Table 9. The temperature difference between the surface and interior refers to the maximum temperature difference between the depths of 5 cm and 50 cm below the concrete surface [4]. From Table 9, it can be seen that the internal maximum temperature and the temperature difference between the surface and interior calculated according to the Chinese code are higher than those obtained using the U.S. code, with the maximum differences being 1.31 °C and 1.25 °C, respectively. This indicates that the temperature control requirements in the Chinese code are more stringent than those in the U.S. code.

#### 4.1.2. Temperature–Time History Curve Analysis of Key Points

**Figure 13:**
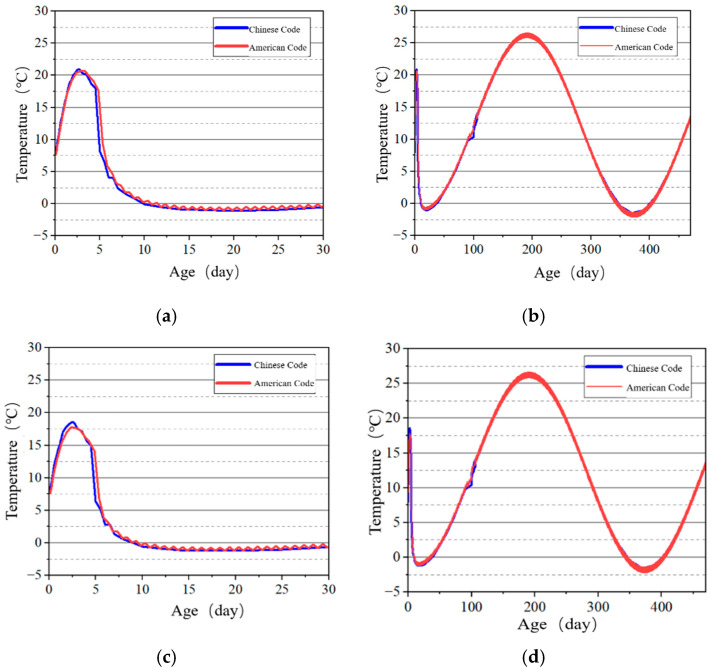
Temperature–time curves of surface key points on the base slab. (**a**) Early age of Point A; (**b**) construction period of Point A; (**c**) early age of Point B; (**d**) construction period of Point B.

**Figure 14:**
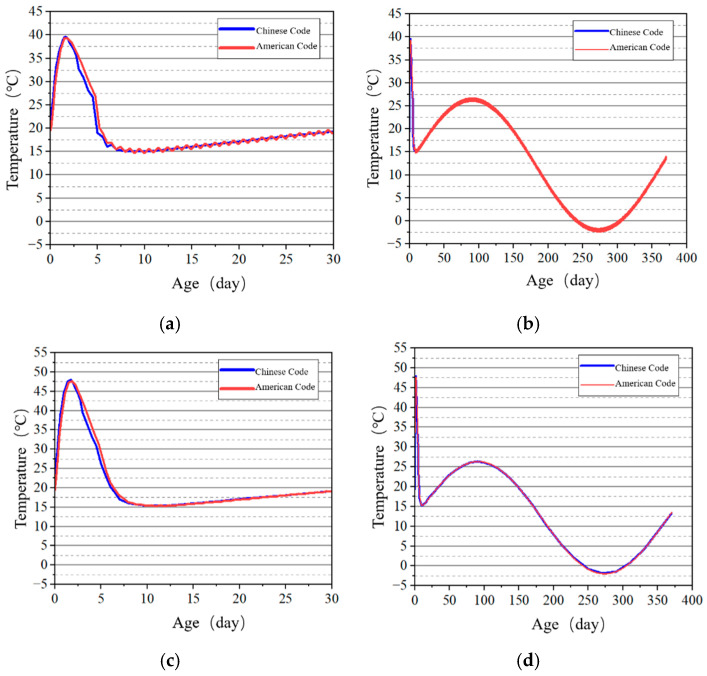
Temperature–time curves of key points on the tidal barrier slot. (**a**) Early age of Point C; (**b**) construction period of Point C; (**c**) early age of Point D; (**d**) construction period of Point D.

**Figure 15:**
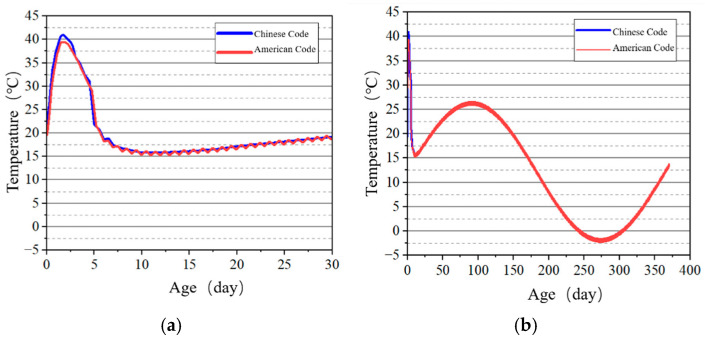
Temperature–time curves of key points on the gate pier surface. (**a**) Early age of Point E; (**b**) construction period of Point E.

**Figure 16:**
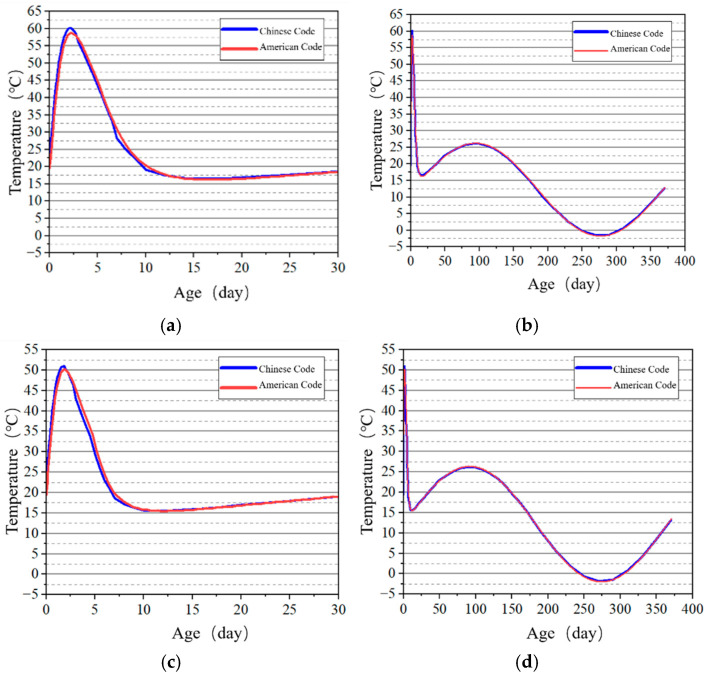
Temperature–time curves of key points on the pier at the X = 0 m section. (**a**) Early age of Point F; (**b**) construction period of Point F; (**c**) early age of Point G; (**d**) construction period of Point G.

Selected surface and internal points of the tidal sluice gate model were used as key points to study the temperature development patterns, and the temperature–time curves shown in Figure 13, Figure 14, Figure 15 and Figure 16 were obtained. The results indicate that the highest temperatures calculated using the Chinese code are slightly higher than those calculated using the U.S. code. However, the overall differences are minimal, and the temperature change trends are consistent. Both codes accurately reflect the temperature field changes during the early curing period of the tidal sluice gate and throughout the entire calculation duration.

**Figure 17:**
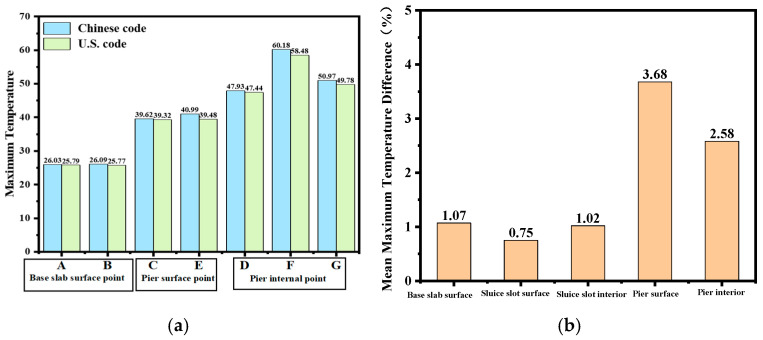
Maximum temperature difference. (**a**) Comparison of maximum temperatures; (**b**) average maximum temperature difference.

Further, a comparison of the maximum temperatures at each key point calculated using different standards is presented in Figure 17. It can be seen that the maximum temperatures calculated using the Chinese code are higher than those calculated using the American code, with differences ranging from 0.24 °C to 1.7 °C. Among them, the surface point of the base slab shows the smallest difference (0.75%), while the temperature difference is largest at key Point E on the gate pier surface, reaching 3.68%.

Considering the calculation results in Table 9, it can be concluded that the maximum temperature and temperature difference between the surface and interior under the Chinese code are higher than those under the American code. This can be attributed to the different calculation functions for the surface heat dissipation coefficient in the two codes. The average wind speed in Weihai City, where the sluice gate project is located, is 3.6 m/s. When the concrete surface is in direct contact with air, the surface heat dissipation coefficients under the Chinese and American codes, calculated according to the formula in Figure 2, are 76.1 kJ/(m^2^·d·°C) and 71.35 kJ/(m^2^·d·°C), respectively. Since the value of the surface heat dissipation coefficient is higher in the Chinese code, the temperature difference between the surface and interior under the Chinese code are higher than those under the American code. The difference in the maximum temperature of the structure can be attributed to calculation errors, either due to the time step or other factors. Overall, the results indicate that the temperature control standards in the Chinese code are more rigorous, resulting in higher structural temperatures and a greater temperature difference between the surface and interior.

### 4.2. Comparison of Stress Field Calculation Results

#### 4.2.1. Stress Field Contour Plot

**Figure 18:**
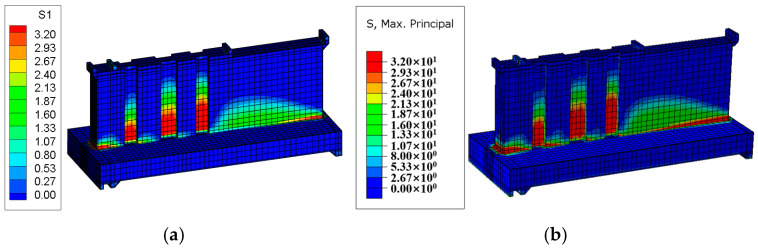
Stress field diagram of the tidal barrier gate after 290 days of pier casting. (**a**) Chinese code; (**b**) U.S. code.

**Figure 19:**
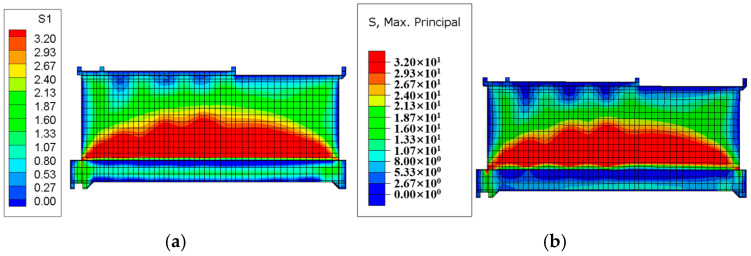
Stress field diagram of the X = 0 m section after 250 days of pier casting. (**a**) Chinese code; (**b**) U.S. code.

**Figure 20:**
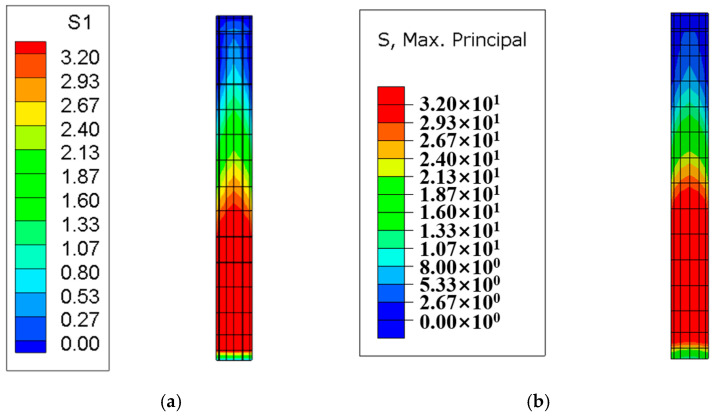
Stress field diagram of the Y = 10 m section after 250 days of pier casting. (**a**) Chinese code; (**b**) U.S. code.

**Figure 21:**
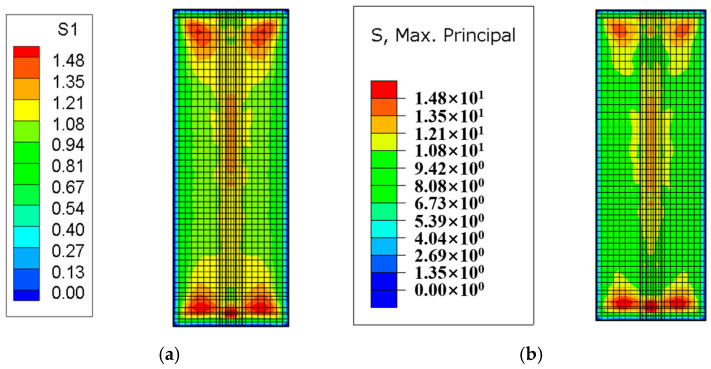
Stress field diagram of the Z = 0.95 m section after 450 days of base slab casting. (**a**) Chinese code; (**b**) U.S. code.

After the temperature field calculation, a finite element simulation of the stress field for the tidal barrier gate was conducted, with tensile stress defined as positive and compressive stress as negative. Typical moments and sections from the finite element model were selected to compare the maximum tensile stress and its distribution under both the Chinese and American codes. The resulting stress field diagrams for the surface and interior of the tidal barrier structure are shown in Figure 18, Figure 19, Figure 20 and Figure 21.

Figure 18 shows the stress field contour map of the gate pier 290 days after pouring. As can be seen from Figure 18, the areas where the surface tensile stress of the gate pier exceeds the standard are mainly concentrated on the surface of the gate slot and the junction between the gate pier and the base slab. This is primarily caused by the significant temperature difference between the upper and lower layers, due to the base slab being poured in winter and the gate pier being poured in spring. The maximum surface tensile stress of the gate pier calculated according to the Chinese and American codes are 4.63 MPa and 5.15 MPa, respectively, while the overall maximum tensile stress of the gate pier is 9.48 MPa and 9.82 MPa, respectively. Under both the Chinese and American codes, the tensile stress of the gate pier exceeds the tensile strength limit of C35 concrete, indicating the need for temperature control and crack prevention measures. Furthermore, it can be observed that the maximum surface tensile stress and overall maximum tensile stress of the gate pier calculated according to the Chinese code are 0.52 MPa and 0.34 MPa lower than those calculated using the American code, respectively. Figure 19, Figure 20 and Figure 21 show the stress field contour maps of the cross-sections at X = 0 m, Y = 10 m, and Z = 0.95 m, respectively, and the maximum tensile stress of each typical cross-section according to both codes is shown in Table 10. From the stress field contour map calculation results, it can be observed that the stress distribution patterns calculated under both codes are consistent, with the maximum tensile stress occurring in the same regions. However, the maximum tensile stress of the structure calculated under the American code is higher than that under the Chinese code, with the difference ranging from 0.21 MPa to 0.52 MPa.

#### 4.2.2. Stress–Time History Curve Analysis of Key Points

**Figure 22:**
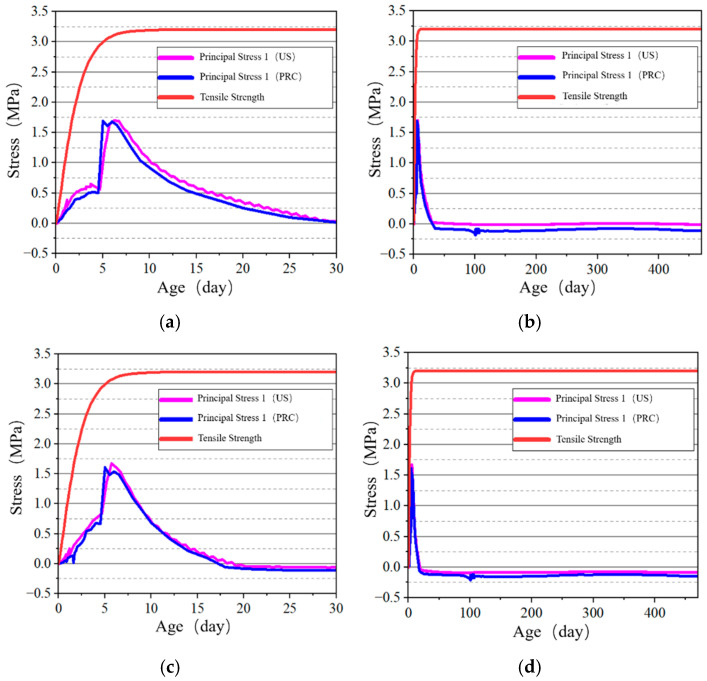
Stress–time curves of surface key points on the base slab. (**a**) Point A early-age period; (**b**) Point A construction period; (**c**) Point B early-age period; (**d**) Point B construction period.

**Figure 23:**
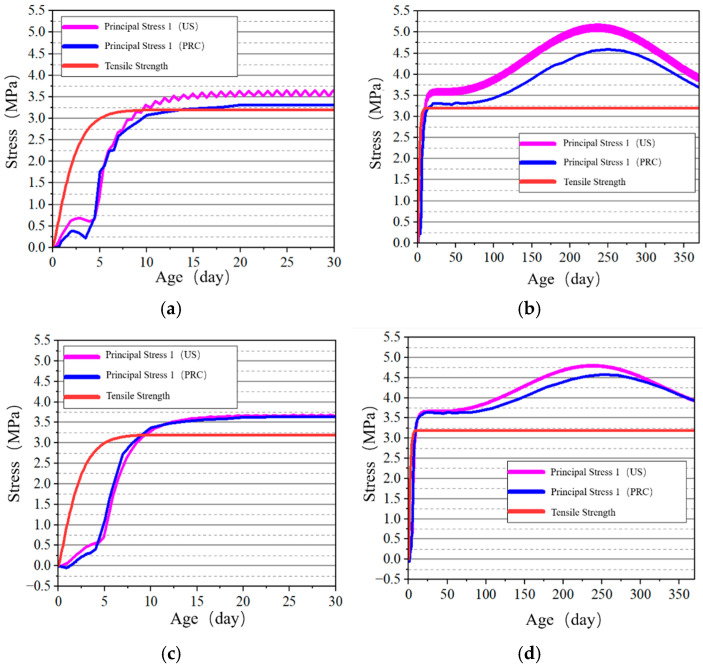
Stress–time curves of surface and internal key points on the gate slot. (**a**) Point C early-age period; (**b**) Point C construction period; (**c**) Point D early-age period; (**d**) Point D construction period.

**Figure 24:**
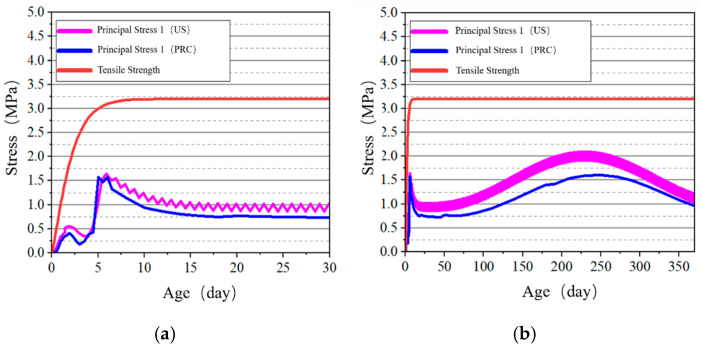
Stress–time curves of surface key points on the pier. (**a**) Point E early-age period; (**b**) Point E construction period.

**Figure 25:**
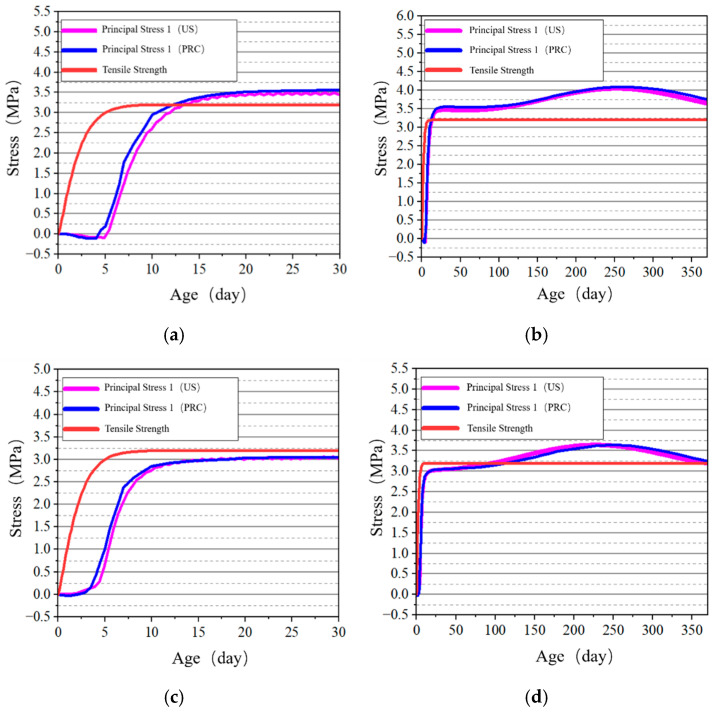
Stress–-time curves of key points on the pier at X = 0 cross-section. (**a**) Point F early-age period; (**b**) Point F construction period; (**c**) Point G early-age period; (**d**) Point G construction period.

The stress development at the surface and internal key points of the tidal barrier gate model was studied, and the stress–time curves are shown in Figure 22, Figure 23, Figure 24 and Figure 25.

**Figure 26:**
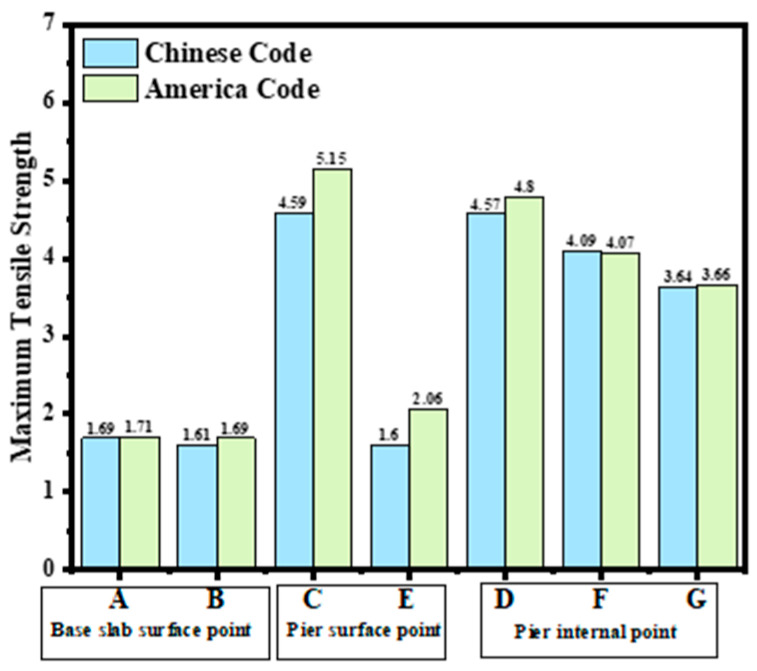
Comparison of maximum tensile stress at key points.

From Figure 22 and Figure 24, it can be observed that tensile stress exceeding the allowable limits occurs at characteristic points located on the surface and interior of the gate slot, as well as at the pier structure. Appropriate temperature control and crack prevention measures are required for these regions. In Figure 23, the tensile stress at the pier gradually decreases after reaching its peak. The stress calculated under the American code exhibits significant fluctuations with ambient temperature changes, while the stress calculated under the Chinese code shows less fluctuation, due to an increase in time step size after a concrete age of 7 days. Figure 22, Figure 23, Figure 24 and Figure 25 demonstrate that the stress evolution patterns of characteristic points are similar under both codes. However, the stresses calculated under the American code are generally higher than those calculated under the Chinese code. The comparison of the maximum tensile stresses at characteristic points under the two codes is shown in Figure 26. It can be observed that, except for the internal key Point F on the pier, the maximum tensile stresses calculated by the American standard are generally higher than those calculated by the Chinese standard, with differences ranging from 0.02 to 0.56 MPa. The variation in maximum tensile stress is smaller for internal key points on the pier, while it is more pronounced for surface key points on the pier, with differences of 0.56 MPa and 0.46 MPa observed at key Points C and E, respectively.

In conjunction with the calculation results of the stress field contour maps, it can be observed that the tensile stress calculated under the American code is higher than that under the Chinese code, with a maximum difference of 0.56 MPa. This discrepancy is primarily attributed to differences in the treatment of creep effects between the two codes. In the Chinese code, the equivalent elastic modulus is a function of the elastic modulus at the time of loading and the creep coefficient of concrete. In contrast, the American code incorporates not only the elastic modulus and creep coefficient but also introduces the concept of the aging coefficient. The value of the aging coefficient is influenced by factors such as the time of loading, the duration of sustained loading, the geometric shape of the loading specimen, and other parameters. At the initial loading time, the theoretical value of the aging coefficient is 0.5, which gradually approaches 1.0 over time. According to Equation (5), the inclusion of the aging coefficient leads to an increased creep coefficient of concrete under the American code, thereby resulting in greater tensile stress. Consequently, the higher tensile stress computed under the American code indicates its higher sensitivity in predicting potential tensile stress risks. Even when the actual stress may not yet reach critical levels, the American code demands precautionary measures to reduce tensile stress based on its elevated calculation results, thereby reflecting a higher safety margin.

## 5. Conclusions

This study, based on a tidal barrier gate in China, conducts a finite element analysis of the temperature and stress fields during the construction of this hydraulic mass concrete structure using temperature control simulation programs from both Chinese and American standards. Surface and internal key points of the gate structure were selected to examine temperature and stress development patterns. The following conclusions were drawn from the analysis.

(1) The temperature field calculation results show that the temperature difference between the interior and exterior of the structure calculated according to the Chinese code is higher than that calculated according to the U.S. code, with a difference range of 0.18 °C to 1.25 °C. This discrepancy is mainly attributed to the higher surface heat dissipation coefficient in the Chinese code, which results in a greater temperature gradient. Additionally, the maximum temperature of the structure calculated under the Chinese code is also higher than that under the U.S. code. The temperature field calculations indicate that the Chinese code exhibits greater sensitivity in predicting high-temperature risks, and has a greater safety margin, providing stricter temperature control guidelines for mass concrete projects with potential high-temperature hazards.
(2) Compared with the Chinese code, the American code incorporates the concept of the aging coefficient in its creep calculation model. This coefficient increases the creep of concrete, thereby indirectly raising the tensile stress of the structure. The stress field calculations show that the maximum tensile stress of the structure under the American code are higher than those under the Chinese code, with an increase ranging from 0.02 to 0.56 MPa. This suggests that the American code places greater emphasis on the potential impact of long-term concrete creep on structural performance.
(3) The temperature and stress field calculations under both codes exhibit similar overall trends. Both codes effectively capture the variations in the temperature and stress fields of the water gate during the early-age period and throughout the entire calculation duration. These findings provide safer and more cost-effective temperature control design solutions for international engineering projects. Furthermore, the results serve as foundational data to support the optimization of national codes and the future development of more unified and universally applicable international standards.

## Figures and Tables

**Table 1:** Common creep models under Chinese and American standards.

	Calculation Formula	
Chinese Standard [4]	ϕ(t,τ)=C(t,τ)·E(τ);C(t,τ)=C1(1+9.20τ−0.45)1−e−0.30(t−τ)+C2(1+1.70τ−0.45)1−e−0.005(t−τ);E(τ)=E01−e−0.4τ0.34;C1=0.23E0;C2=0.52E0	(2)
American Standard (ACI 209) [22]	$\left\{\begin{array}{l} J(t,t_{o})=\frac{1+\phi (t,t_{o})}{E_{cmto}};E_{cmto}=0.043\gamma_{c}^{1.5}\sqrt{f_{cmto}};\\ f_{cmt}=\left[\frac{t}{a+bt}\right]f_{cm28};\phi (t,t_{o})=\frac{{\left(t-t_{o}\right)}^{\Psi }}{d+{\left(t-t_{o}\right)}^{\Psi }}\phi_{u} \end{array}\right.$	(3)
American Standard (B3) [22]	$\left\{\begin{array}{l} J(t,t_{o})=q_{1}+C_{O}(t,t_{o})+C_{d}(t,t_{o},t_{c});q_{1}=0.6/E_{cm28};\\ C_{O}(t,t_{o})=q_{2}Q(t,t_{o})+q_{3}\cdot \ln\left[1+{\left(t-t_{o}\right)}^{n}\right]+q_{4}\cdot \ln(t/t_{o});\\ C_{d}(t,t_{o},t_{c})=q_{5}{\left[\exp\left\{-8H(t)\right\}-\exp\left\{-8H(t_{o})\right\}\right]}^{1/2} \end{array}\right.$	(4)
American Standard (GL 2000) [22]	$\left\{\begin{array}{l} \phi 28(t,t_{o})=\frac{1}{\chi (t,t_{0})}\cdot C(t,\tau )\cdot E(\tau );\\ E_{cmt}=3500+4300\sqrt{f_{cmt}}\\ f_{cmt}={\beta_{e}}^{2}f_{cm28}\\ f_{cm28}=1.1f_{c}^{\prime }+5.0 \end{array}\right.$	(5)

**Table 2:** Common adiabatic temperature rise models under Chinese and American standards.

	Calculation Formula	
Chinese standard (Exponential model) [4]	$\theta (\tau )=\theta_{0}(\tau -e^{-m\tau })$	(6)
Chinese standard (Hyperbolic model) [4]	$\theta (\tau )=\theta_{0}\tau /(n+\tau )$	(7)
Chinese standard (Composite exponential model) [4]	$\theta (\tau )=\theta_{0}(1-e^{-a\tau^{b}})$	(8)
American standard (Maximum Allowable Temperature Difference Method) [23]	$\Delta T=\frac{\varepsilon_{tsc}}{K\alpha_{c}R}$	(9)
American standard (PCA Method) [23]	$T_{\max}=T_{i}+(12\cdot \frac{W_{c}}{100})+(6\cdot \frac{W_{scm}}{100})$	(10)
American standard (Schmidt Temperature Gradient Method) [23]	$\left\{\begin{array}{l} \frac{d}{dx}\left(k\cdot \frac{dT}{dx}\right)+\frac{d}{dy}\left(k\cdot \frac{dT}{dy}\right)+Q_{H}=\rho \cdot C_{p}.\frac{dT}{dt}\\ \Delta t=\frac{\left(\Delta x^{2}\right)}{2\alpha } \end{array}\right.$	(11)

**Table 3:** Hydration heat constants for cement types.

Cement Type	a	b
Ordinary Portland Cement 425#	0.69	0.56
Ordinary Portland Cement 525#	0.36	0.74
Slag Portland Cement for Dams 425#	0.29	0.76
Ordinary Portland Cement for Dams 525#	0.79	0.70

**Table 4:** Hydration heat of different fineness of cement.

Cement Type	Fineness (cm^2^/g)	Heat of Hydration Calories per g
I	1790	87
Ⅱ	1890	76
Ⅲ	2030	106
Ⅳ	1910	60

**Table 5:** Foundation and concrete thermophysical and mechanical properties.

Material		Thermal Conductivity (kJ/(m· h·°C))	Specific Heat (kJ/(kg·°C))	Thermal Diffusivity (m^2^/h)	Linear Expansion Coefficient (10^−6^/°C)	Poisson’s Ratio	Density (kg/m^3^)	Young’s Modulus (GPa)
Foundation	Soil	2.41	1.91	0.0012	0.80 × 10^−5^	0.22	1830	0.1
	Rock	10.50	0.81	0.0055	0.85 × 10^−5^	0.19	2680	20
Concrete	C15	9.663	0.924	0.00445	8.6 × 10^−6^	0.167	2350	
	C30	9.465	0.913	0.00439	9.0 × 10^−6^	0.167	2360	
	C35	9.381	0.902	0.00437	9.5 × 10^−6^	0.167	2381	

**Table 6:** Annual average temperature of Weihai city.

Month	1	2	3	4	5	6	7	8	9	10	11	12
Measured values (°C)	−3	−1.4	5.8	11.5	17.2	21.8	25.9	27.2	20.5	13.2	7.5	0

**Table 7:** Sluice gate pouring schedule.

No.	Name	Schedule	Initial Temperature (°C)
1	Cushion	12.20	8
2	Base plate	12.30	8
3	Gate pier	4.10	19.5

**Table 8:** Key points’ locations.

Key Points	A	B	C	D	E	F	G
X	2.95	3.75	0.35	0.00	0.75	0.00	0.00
Y	9.73	8.25	9.73	9.73	12.93	13.87	3.10
Z	2.00	1.47	3.38	3.86	2.90	3.38	3.38

**Table 9:** Internal maximum temperature and temperature difference between the surface and interior for typical sections.

Section	Internal Maximum Temperature		Temperature Difference Between Surface and Interior	
	Chinese Code	American Code	Chinese Code	American Code
X = 0 (1 day after pier pouring)	56.16	55.93	17.55	17.37
Y = 10 (1 day after pier pouring)	47.52	46.21	8.68	7.43
Y = 10 (5 days after slab pouring)	33.16	32.79	19.18	18.84
Z = 0.95 (5 days after slab pouring)	34.16	33.48	20.44	19.80

**Table 10:** Maximum tensile stress of typical sections under Chinese and American codes.

Section	Maximum Tensile Stress (MPa)		
	Chinese Code	U.S. Code	Difference
X = 0	6.95	7.39	0.44
Y = 10	4.94	5.20	0.26
Z = 0.95	1.62	1.83	0.21

**Figure A1:**
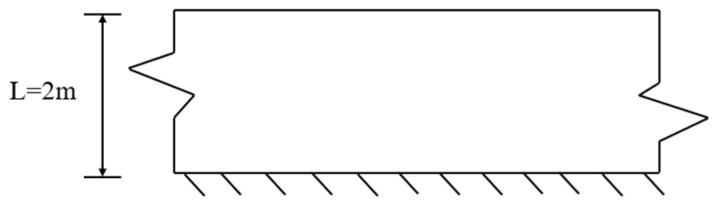
Schematic of the infinitely large slab.

**Figure A2:**
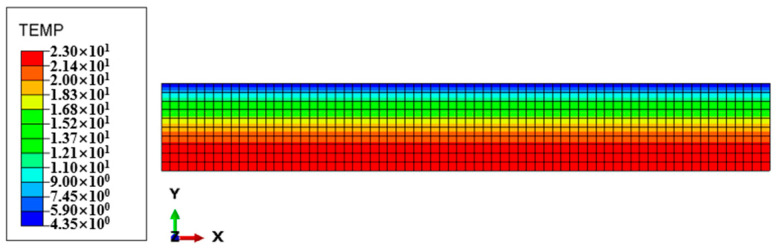
Temperature field contour of the slab after 7 d of pouring (unit: °C).

**Figure A3:**
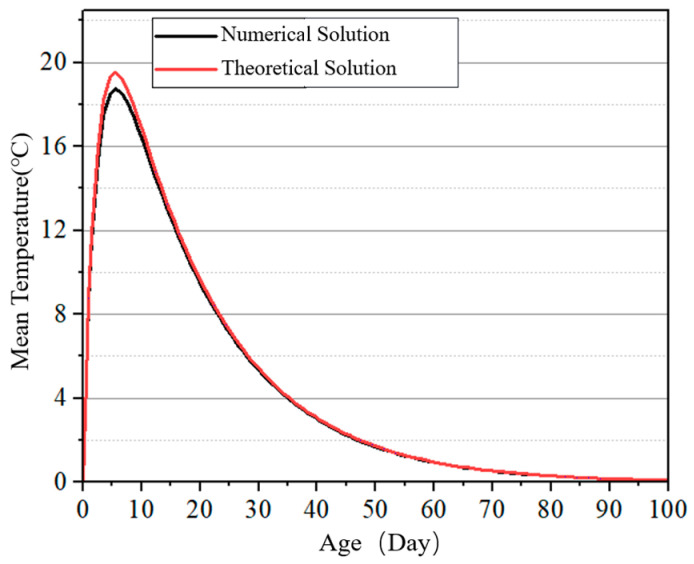
Comparison of theoretical and numerical solutions for average slab temperature.

**Figure A4:**
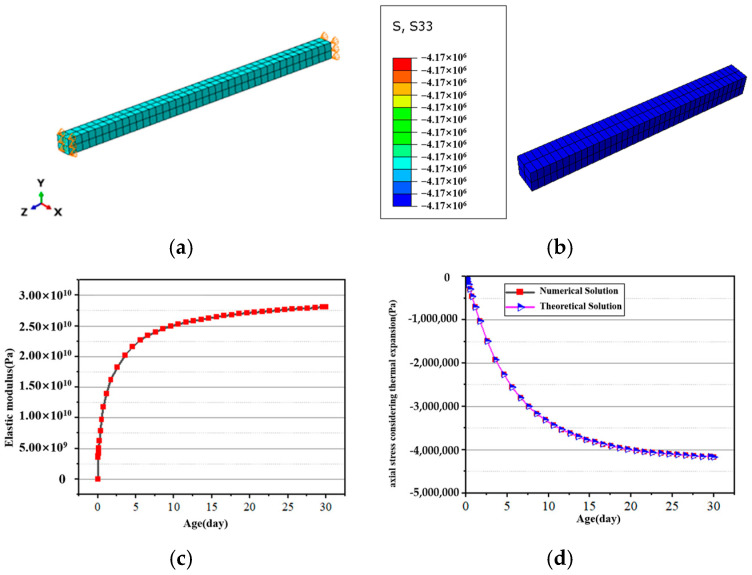
Stress verification of a rod considering thermal expansion. (**a**) Rod with constraints applied at both ends; (**b**) axial stress S33 of the rod considering thermal expansion; (**c**) elastic modulus of the rod; (**d**) comparison of numerical and theoretical solutions for axial stress.

**Figure A5:**
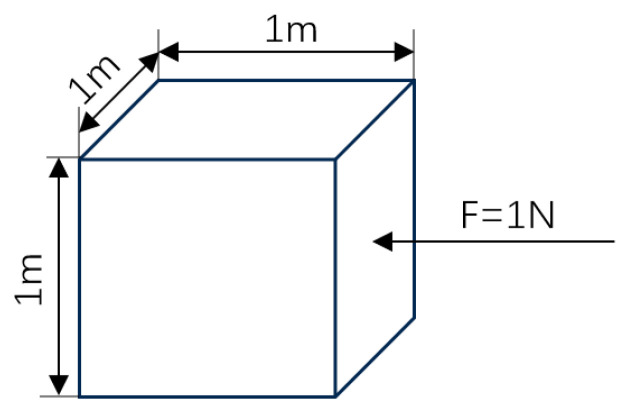
Uniaxial compression of the cubic model.

**Figure A6:**
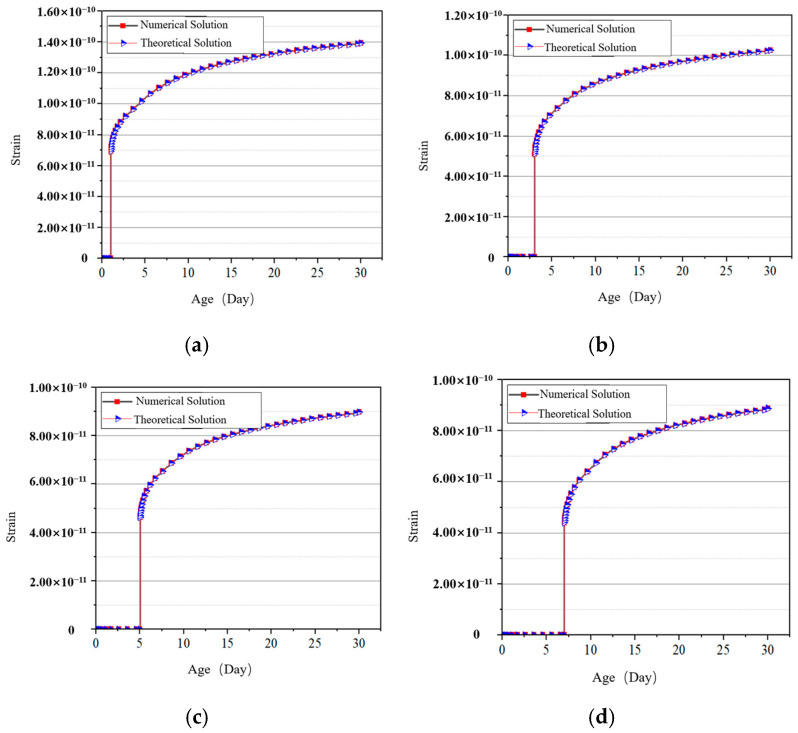
Comparison of finite element numerical solution and theoretical solution for concrete creep. (**a**) Loading age of 1 day; (**b**) loading age of 3 days; (**c**) loading age of 5 days; (**d**) loading age of 7 days.

## Data Availability

The original contributions presented in this study are included in the article. Further inquiries can be directed to the corresponding author.

## Appendix A

1. ABAQUS Development Environment Setup

In this study, Fortran is used for secondary development of ABAQUS subroutines. The following combinations of ABAQUS versions with vs. and IVF are recommended.

**Table A1:** Vs. and IVF combinations associated with ABAQUS.

ABAQUS Installation Version	Compatible IVF Versions	Compatible vs. Versions
6.13	V10.1, v11.0, v11.1 and above	2008, 2010, 2012
6.14	2011, 2013	2010, 2012, 2013
2016	2013	2012, 2013
2017	2013	2012, 2013
2018, 2019, 2020	2016	2015

2. Temperature Field Subroutine Verification

In engineering practice, the construction thickness of concrete is typically much smaller than its entire plane dimensions, allowing for the simplification of concrete heat dissipation behavior to one-dimensional heat transfer. Therefore, this study selects an infinitely large two-dimensional slab as the calculation model for verifying the temperature field subroutine. The slab has a thickness of 2 m, with an initial temperature of 0 °C. The bottom and side surfaces are in an adiabatic state, while the top surface dissipates heat. The ambient temperature is set to 0 °C, with a concrete density of 2400 kg/m^3^, a surface heat transfer coefficient of β = 1200 kJ/(m^2^·d·°C), a thermal conductivity λ = 240 kJ/(m· d·°C), and a thermal diffusivity of 0.2 m^2^/d. A schematic of the infinitely large two-dimensional slab model is shown in Figure A1.

Using the HETVAL and FILM subroutines, we simulated the temperature field of the infinitely large slab. The temperature field contour plot of the concrete slab after 7 d of pouring is shown in Figure A2.

A comparison of the finite element numerical solution and theoretical solution of the average temperature in the slab is shown in Figure A3.

According to Figure A3, the theoretical and numerical solutions for the average slab temperature show a similar trend over time, with slight deviations near the temperature peak. Typical time points after pouring were selected to compare the error between the numerical and theoretical solutions for the average temperature at different moments, as shown in Table A2.

**Table A2:** Average temperature and error of the slab at different time points. (unit: °C).

Age	1 d	3 d	5 d	7 d	14 d	28 d	56 d	90 d
Numerical Solution of Temperature	8.62	15.35	18.46	18.58	13.71	6.18	1.23	0.173
Theoretical Solution of Temperature	8.74	15.94	19.24	19.27	14.01	6.29	1.25	0.177
Error	0.12	0.59	0.78	0.69	0.3	0.11	0.02	0.004
Relative Error	1.37%	3.70%	4.05%	3.58%	2.14%	1.74%	1.60%	2.25%

As shown in Table A2, the maximum difference in average temperature for the infinitely large two-dimensional slab is 0.78 °C, and the maximum relative error between the numerical and theoretical solutions for the average temperature is 4.05%. This maximum relative error falls within the acceptable range of 5%, indicating that the HETVAL and FILM subroutines developed in this study based on ABAQUS software(version 2023) can effectively simulate the temperature field behavior of large-volume concrete under the combined effects of hydration heat and ambient temperature.

3. Stress Field Subroutine Verification

(1) Stress Analysis of a Rod Considering Thermal Expansion

A concrete rod constrained at both ends is considered. The rod length L is 1 m, with a cross-sectional size of b × h = 0.05 m × 0.05 m. The mesh size for the rod is set to 0.025 m. The ambient temperature is 0 °C, with an initial temperature of 15 °C. The thermodynamic parameters of the rod are detailed in Table A3.

**Table A3:** Thermodynamic parameters of the rod.

Indicator	Value	Unit
Density	2400	kg/m^3^
Average Compressive Strength	fcm28=30	MPa
Poisson’s Ratio	0.167	
Linear Expansion Coefficient	0.000008	10^−6^/°C
Thermal Conductivity	9.18	kJ/(m h·°C)
Specific Heat	0.887	kJ/(kg·°C)
Hydration Temperature Rise Model	$\theta (\tau )=30(1-e^{-0.36\tau^{0.74}})$	°C
Young’s Modulus	$\beta_{e}=\exp\left[\frac{s}{2}\left(1-\sqrt{\frac{28}{t}}\right)\right]$ $f_{cmt}={\beta_{e}}^{2}f_{cm28}$ $E_{cmt}=3500+4300\sqrt{f_{cmt}}$	MPa

In Table A3, $E_{cmt}$ represents the elastic modulus of concrete in MPa, t represents the age of the concrete in days, and s is a coefficient related to the type of cement. For ordinary cement, the value of s is set to 0.335. When considering the heat of hydration of the concrete rod, the calculated stress field results are shown in Figure A4 (version 2023). Thermal stress is calculated as: S=ΔT∗∂∗Et. ΔT represents temperature increment, °C; ∂ represents the coefficient of thermal expansion, 1/°C; and Et represents elastic modulus, Pa.

As shown in Figure A4, the numerical solution for the rod’s axial stress due to thermal expansion aligns closely with the theoretical solution. The UMAT subroutine provides an accurate simulation of temperature-induced stress resulting from concrete thermal expansion.

(2) Uniaxial Compression

A 1 m^3^ concrete specimen was selected as the study object to validate the creep constitutive model of concrete. As shown in Figure A5, a constant axial force of F = 1 N is applied to the specimen at time t_0_, with a fixed boundary constraint applied to the opposing face.

Finite element calculations yielded both numerical and theoretical strain solutions for the concrete specimen at various loading times, as shown in Figure A6. From Figure A6, it can be observed that the theoretical and numerical strain solutions of the cubic concrete specimen are in close agreement across the four different loading ages, confirming the effectiveness of the viscoelastic creep constitutive model in the UMAT subroutine.
